# Combined Manganese-Iron Exposure Reduced Oxidative Stress is Associated with the NRF2/NQO1 Pathway in Astrocytic C8-D1A Cells

**DOI:** 10.1007/s12011-025-04708-9

**Published:** 2025-06-17

**Authors:** Maximus Wong, Aafia Ahmed, Wenjing Luo, Aaron B. Bowman, Yousef Tizabi, Michael Aschner, Beatriz Ferrer

**Affiliations:** 1 Department of Molecular Pharmacology, Albert Einstein College of Medicine, Jack and Pearl Resnick Campus, Forchheimer Building, 1300 Morris Park Avenue, Bronx, NY 10461 USA; 2 Department of Occupational and Environmental Health, The Ministry of Education Key Lab of Hazard Assessment and Control in Special Operational Environment, School of Public Health, Fourth Military Medical University, Xi’an, China; 3 School of Health Sciences, Purdue University, West Lafayette, IN 47907 USA; 4 Department of Pharmacology, Howard University College of Medicine, Washington, DC 20059 USA

## Abstract

Manganese (Mn) and iron (Fe) are essential trace metals. Both are essential for multiple physiological processes, including brain function, metabolism, and cellular respiration. However, excessive exposure to these metals can have detrimental health effects, particularly in occupational exposures, such as mining, welding, battery production, and iron and steel manufacturing. Mn and Fe accumulate in astrocytes, especially in brain regions involved in motor control and cognition, such as the substantia nigra and globus pallidus in the basal ganglia. Excessive exposure to Mn and Fe induces oxidative stress, neuronal damage and neurodegeneration, and has been implicated in various neurodegenerative diseases, including Alzheimer’s disease (AD) and Parkinson’s disease (PD). Here, we investigated the effects of combined Mn and Fe exposure on C8-D1A astrocytic cells and explored the associated oxidative stress pathways. Our results demonstrated that Mn exposure decreased Superoxide dismutase 2 (*Sod2) mRNA* expression and one of its upstream regulators, Signal Transducer and Activator of Transcription 3 (STAT3) protein and gene levels, associated with an increase in oxidative stress, whereas Fe exposure had no effect on this pathway. Interestingly, combined Mn and Fe exposure decreased reactive oxygen species (ROS) levels and upregulated the expression of the antioxidant gene NAD(P)H quinone dehydrogenase 1 (NQO1) compared to Mn and Fe exposure alone. Our findings suggest that combined Mn and Fe exposure activate the Nuclear factor erythroid 2-related factor 2 (NRF2)/NQO1 antioxidant signaling pathway in C8-D1A astrocytic cells, mitigating oxidative stress and protecting cells from damage. By understanding these mechanisms, novel therapeutic targets for neurodegenerative diseases associated with occupational metal exposures may be identified.

## Introduction

Manganese (Mn) and iron (Fe) are abundant elements in the environment. While essential for various physiological processes, excessive exposure to these metals can have detrimental health consequences [1]. Typically, adults consume 12–18 mg of Fe and 1.8–2.3 mg of Mn daily through their diet [2]. However, occupational exposures (e.g., welding) and some diets, can lead to chronic exposure to these metals [1]. This prolonged exposure may result in the accumulation of Mn and Fe within the brain, potentially exacerbating neurotoxicity.

Mn is an essential trace element involved in various physiological functions, such as brain function, nerve and brain development, and metabolism [3–5]. Legumes, nuts, whole grains, seeds, seafood, chocolate, tea, and some fruits and vegetables are dietary sources of Mn [6]. It is also present in industrial products such as fireworks, fertilizers, paints, and cosmetics [4]. While essential in small amounts, Mn deficiency, a rare physiological condition, can have adverse health effects, including impaired fertility, glucose intolerance, skeletal abnormalities, and growth retardation [7].

Excessive Mn exposure can have detrimental effects on the central nervous system (CNS). Mn can accumulate in the brain, particularly within astrocytes in regions enriched with dopaminergic neurons, such as the substantia nigra [8, 9]. This accumulation can result in dopamine oxidation and neuronal damage, causing alterations in brain function and behavior, including cognitive impairment, mood disorders, and motor disturbances [3, 10]. Chronic exposure to Mn can result in manganism, a neurological condition characterized by Parkinsonian-like symptoms, such as tremors, rigidity, bradykinesia, and postural instability [11]. Manganism results from a deficiency in dopamine neurotransmission caused by the degeneration of dopaminergic neurons in the basal ganglia [12, 13].

Mn-induced toxicity in cells is often linked to the production of reactive oxygen species (ROS) [14, 15], which may result from the disruption of mitochondrial function [16], the primary source of cellular energy. Furthermore, Mn can impair antioxidant defense systems by interfering with glutathione (GSH) synthesis [17], intensifying oxidative stress and contributing to neurotoxicity. In addition to oxidative stress, Mn can disrupt glutamate homeostasis [18–20]. By interfering with glutamate uptake by astrocytes, Mn contributes to elevated extracellular glutamate levels, and ensuing excitotoxicity, triggering excessive sodium and calcium influx, further amplifying ROS production.

Fe is another essential trace element required for numerous biological processes, including energy production, oxygen transport, and cellular respiration [1, 21]. Like Mn, Fe is necessary for various enzymatic functions, particularly within the CNS, where it plays a pivotal role in neural activity [22]. Maintaining Fe balance is critical, as both deficiency and overload can have detrimental effects on the CNS. Fe deficiency impairs brain development and function [23], while excessive Fe exposure can contribute to neurodegeneration [24], particularly in the substantia nigra pars compacta of the basal ganglia, leading to the degeneration of dopaminergic neurons and a subsequent reduction in dopamine levels, contributing to PD [25]. Primary sources of Fe overload in humans include excessive dietary intake, genetic hemochromatosis, and occupational exposures such as welding, iron and steel production, and foundry work.

Astrocytes play a critical role in maintaining Fe homeostasis within the brain [26–28]. By regulating Fe uptake, storage, and transport, astrocytes act as a buffer in maintaining Fe homeostasis and therefore prevent Fe-induced neurotoxicity. However, when Fe overload occurs in astrocytes, it can contribute to neurodegeneration by triggering oxidative stress and mitochondrial dysfunction, key factors in the pathogenesis of various neurological disorders [29, 30]. Fe can participate in Fenton reactions, generating ROS that promote cellular damage [1, 31] Furthermore, Fe overload also leads to mitochondrial dysfunction, exacerbating the cellular toxicity induced by ROS [1, 31], and inhibits the antioxidant defense system by depleting GSH levels and reducing nicotinamide adenine dinucleotide phosphate (NADPH), thereby amplifying the effects of oxidative stress [32], similar to the effects of Mn.

Mn and Fe share overlapping chemical properties, including their distribution within the brain and their utilization of shared transport mechanisms [33]. Mn and Fe, in their divalent forms, are transported into the cells by divalent metal transporters, such as Divalent Metal Transporter 1 (DMT1) [34]. Ferroportin 1 (Fpn1) is the only known Fe export protein [35, 36], and it also mediates Mn efflux from cells [37]. In their trivalent form they can bind transferrin (Tf), and this complex crosses the blood–brain barrier (BBB) primarily through the transferrin receptor (TfR) [38]. These shared characteristics contribute to a complex interplay between Mn and Fe homeostasis. Animal studies have shown that Fe deficiency increases Mn absorption [39–41] and human studies corroborate these findings, with iron-deficient individuals exhibiting higher blood Mn levels [42–44]. Importantly, Fe deficiency exacerbates the negative effects of Mn exposure on neurodevelopment. Studies have shown that, children of mothers with low blood hemoglobin during pregnancy exhibited stronger negative associations between prenatal Mn exposure and cognitive outcomes at 6 months of age [45]. Similarly, children whose mothers had low prenatal ferritin levels demonstrated poorer memory and lower General Cognitive Index scores when exposed to higher prenatal Mn levels [46]. These findings suggest that Fe status may modify the impact of Mn and Mn may modify Fe impact on neurodevelopment.

Despite significant advancements in environmental protection and a reduction in airborne Mn concentrations below occupational health standards, concerns remain regarding the health of workers exposed to low levels of Mn. Notably, welding and Mn smelting environments may contain high levels of both Mn and Fe [47, 48]. This raises the possibility that simultaneous exposure to Mn and Fe during these occupational settings may synergistically contribute to brain damage, potentially increasing the risk of PD.

Limited research has investigated the neurotoxicity of combined occupational exposure to Mn and Fe. Combined exposure to Mn and Fe may have synergistic effects, potentially exacerbating oxidative stress and contributing to neurodegeneration.

Astrocytes support neurons metabolically by providing essential molecules for energy production and neurotransmitter synthesis, and they also contribute to synapse formation [49]. Compared to neurons, astrocytes have a greater capacity to induce antioxidant defense mechanisms [50–52]. They are among the primary cells in the CNS to activate Nrf2-keap1- ARE, a master antioxidant signaling pathway. This activation enhances their antioxidant capacity and protects neurons by detoxifying ROS and reducing oxidative damage [53–55].

Given the critical role of astrocytes in protecting the CNS from oxidative damage [54], this study aimed to investigate the individual and combined effects of Mn and Fe on oxidative stress and antioxidant mechanisms in C8-D1A astrocytic cells.

## Materials and Methods

### Cell Culture

C8-D1A astrocytic cells were purchased from the American Type Culture Collection (ATCC, USA, CRL-2541). Cells were cultured in Dulbecco’s Modified Eagle Medium (DMEM, Gibco, USA, 11,995,040) supplemented with 10% heat inactivated fetal bovine Serum (FBS, Gibco, USA, A52568-01) and 1% penicillin–streptomycin (Gibco, USA, 15,140–122). Cells were incubated in a humidified incubator at 37 °C with 5% CO_2_.

### Manganese and Iron Treatments

Cells were subcultured in various plates and subsequently treated for 24 h with Manganese (II) Chloride (MnCl_2_, Sigma-Aldrich, USA, 63,535) at concentrations of 0, 50, 200, and 800 μM, Iron (II) Chloride (FeCl_2_, Sigma-Aldrich, USA, 372,870) at concentrations of 0, 100, 500, and 2000 μM, or a combination of both MnCl2 (200 μM) and FeCl2 (100, 500, or 2000 μM), unless otherwise specified.

### Cytotoxicity Assay

Cytotoxicity was assessed with the CyQUANT LDH Cytotoxicity Assay (Invitrogen, USA, C20301), which measures lactate dehydrogenase (LDH) release. Cells were seeded in a 96-well plate and placed at 37 °C with 5% CO_2_ for 24 h. Cells were treated with varying concentrations of Mn, Fe, or a combination of both diluted in phenol red-free DMEM (Gibco, USA, 11,995,040). Following treatment, 50 μL of media from each well was transferred to a new plate and 50 μL of LDH reaction mixture was added to each well. The plate was then incubated for 30 min in the dark. After incubation, 50 μL of stop solution was added, and LDH concentration was determined by measuring absorbance at 490 nm and 680 nm using a SpectraMax iD3 microplate reader (Molecular Devices, USA).

### Cell Viability Assay

Cell viability was assessed using the 3-(4,5-dimethylthiazol-2-yl)−2,5-diphenyltetrazolium bromide (MTT) assay (Invitrogen, USA, M6494). Cells seeded in a 96-well plate were treated with various concentrations of Mn, Fe, or a combination of both Mn and Fe diluted in DMEM (Gibco, USA, 11,995,040) for 24 h. Subsequently, the culture medium was removed, and cells were incubated with 0.5 μg/μL of MTT (dissolved in Hank’s Balanced Salt Solution (HBSS)) for 2 h. Following incubation, the MTT solution was removed, and the formazan crystals were solubilized in dimethyl sulfoxide (DMSO, Sigma-Aldrich, D8418). Absorbance was measured at 540 nm using SpectraMax iD3 microplate reader (Molecular Devices, USA).

### Reactive Oxygen Species (ROS) Measurement

ROS production was measured using the cell-permeable 2',7'-dichlorodihydrofluorescein diacetate (CM-H2DCFDA) probe (Thermo Fisher Scientific, USA, C6827). Cells were seeded in a black 96-well plate and maintained in a humidified incubator with 5% CO_2_ at 37 °C. Afterward, cells were washed with HBSS (Gibco, USA, 14,025–092) and incubated with the CM-H_2_DCFDA probe for 30 min at 37 °C with 5% CO_2_. Following probe incubation, cells were exposed to Mn, Fe, or a combination of both Mn and Fe, diluted in Hanks’ Balanced Salt Solution (HBSS) (Gibco, USA, 14,025–092). Fluorescence intensity was measured at excitation and emission wavelengths of 495 nm and 535 nm, respectively, at the following time points: 30 min, 1 h, 3 h, 6 h, and 24 h.

### Glutathione (GSH) Detection Assay

Cellular GSH levels were determined in cells seeded in 150 mm culture dishes and exposed to 0 or 200 μM Mn, 0 or 500 μM Fe, or a combined treatment of 200 μM Mn and 500 μM Fe. Total GSH levels were quantified using the SensoLyte Total GSH Assay Kit (Anaspec, USA, AS-72153) according to the manufacturer’s instructions. Briefly, cells were collected, centrifugated at 2500 rpm for 5 min, and resuspended in phosphate-buffered saline (PBS). Cells were then lysed by performing three freeze–thaw cycles. Following lysis, cell lysates were deproteinated and centrifuged at 12,000 rpm for 5 min at 4 °C. Protein concentrations in the cell lysates were measured using the Pierce BCA Protein Assay Kit (Pierce, USA, 23,227). Finally, GSH levels were measured at 405 nm using SpectraMax iD3 microplate reader (Molecular Devices, USA).

### Western Blot Analysis

Cells were seeded in 60-mm culture dishes and treated with 0 or 200 μM Mn, 0 or 500 μM Fe, or a combination of 200 μM Mn and 500 μM Fe. Subsequently, cells were lysed using cold radioimmunoprecipitation assay (RIPA) buffer (Pierce, USA, 89,901) supplemented with protease inhibitor cocktails 2 and 3 (Sigma-Aldrich, Israel, P5726-5ML and P0044-1ML, respectively) and Halt™ protease inhibitor cocktail (Thermo Scientific, USA, 1,861,278). Cell lysates were subjected to sonication and then centrifuged at 10,000 × g for 10 min at 4 °C. Protein concentration in the supernatant was determined using the Pierce BCA Protein Assay Kit (Pierce, USA, 23,227). Samples were diluted in Laemmli sample buffer (Bio-Rad, USA, 1,610,737) and boiled at 100 °C for 5 min. For Western blot analysis, equal amounts of protein (20 μg) were loaded onto a Mini-PROTEAN TGX Gel (Bio-Rad, USA, 4,561,096) and separated by SDS-PAGE. Proteins were then transferred to a nitrocellulose membrane. Non-specific binding sites were blocked with 5% bovine serum albumin (BSA, Fisher BioReagents, USA, BP1600-100) in Tris buffer saline containing 0.1% Tween-20 (TBS-T, Promega, USA, H5151) at room temperature. Membranes were then incubated overnight at 4 °C with primary antibodies against pERK (Cell Signaling, USA, 9101), ERK (Cell Signaling, USA, 9102), HO-1 (Cell Signaling, USA, 70,081), NRF2 (Cell Signaling, USA, 12,721), pSTAT3 (Cell Signaling, USA, 9145), STAT3 (Cell Signaling, USA, 12,640), and Actin (Sigma-Aldrich, USA, A1978) diluted in 5% BSA in TBS-T. After washing with TBS-T, membranes were incubated with the appropriate horseradish peroxidase (HRP)-conjugated secondary antibodies. Protein bands were visualized using Image Studio Ver 5.2 (LICORbio, USA) or the ChemiDoc™ Touch Gel Imaging System (Bio-Rad, USA). Band intensities were quantified using ImageJ software (NIH). Protein expression levels were normalized to β-actin levels as an internal loading control.

### RNA Extraction and Real-Time Quantitative PCR

Cells were seeded in 60-mm culture dishes and exposed to 0 or 200 μM Mn, 0 or 500 μM Fe, or a combination of 200 μM Mn and 500 μM Fe. Total RNA was isolated using the Qiagen RNeasy Mini kit (Qiagen, Germany, 74,104) according to the manufacturer’s protocol. RNA concentration and purity were determined using a Nanodrop 2000 Spectrophotometer (Thermo Fisher Scientific, USA, ND2000). Subsequently, RNA samples were reverse transcribed into complementary DNA (cDNA) using the High-Capacity cDNA Reverse Transcription Kit (Applied Biosystems, Lithuania, 4,368,814), following the manufacturer’s protocol.

Quantitative real-time PCR (RT-qPCR) was performed using a CFX96 Real-Time PCR Detection System with the CFX Manager software (Bio-Rad, USA). Gene expression levels were quantified using TaqMan Gene Expression Master Mix (Applied Biosystems, Lithuania, 4,369,016) and normalized to glyceraldehyde 3-phosphate dehydrogenase (*Gapdh*) expression (Mm99999915_g1). Gene expression was calculated using the 2^**–∆∆Ct**^ method [56].

The following TaqMan Gene Expression Assays were purchased from Applied Biosystems: *Hmox1* (Mm00516005_m1), *Nqo1* (Mm01253561_m1), *Slc7a11* (Mm00442530_m1), *Socs3* (Mm00545913_s1), *Sod2* (Mm01313000_m1), and *Stat3* (Mm01219775_m1).

### Statistical Analysis

Statistical analyses were performed using IBM Statistical Package for the Social Sciences (SPSS) version 29.0.2.0(20) software. Graphical data representation was carried out using GraphPad Prism 10.4.1software. No sample size calculations were performed. No test for outliers was conducted, and no data points were excluded. All data were tested for normality using the Shapiro–Wilk test and the Kolmogorov–Smirnov test. The effects of Mn and Fe alone on cytotoxicity, cell viability, and ROS production, were analyzed using one-way analysis of variance (ANOVA), followed by Bonferroni’s post hoc test. When normality was not achieved, the non-parametric Kruskal–Wallis test, followed by Dunn’s post hoc test adjusted by Bonferroni correction, was used.

The effects of combined Mn-Fe exposure on cytotoxicity, cell viability, ROS production, protein expression, gene expression, and GSH levels were assessed using two-way ANOVA, followed by Bonferroni’s post hoc test. F values were indicated as F_Mn_ (Manganese significant effect), F_Fe_ (Iron significant effect), and F_Inf_ (Manganese x Iron interaction significant effect). For data that did not pass normality test, a logarithmic or square root transformation was performed to approximate to a better normal distribution. Data are presented as the mean ± standard deviation (SD). Statistical significance was defined as *p* < 0.05.

## Results

### Manganese Exposure Induced Oxidative Stress Without Cytotoxicity in C8-D1A Astrocytic Cells

**Fig. 1:**
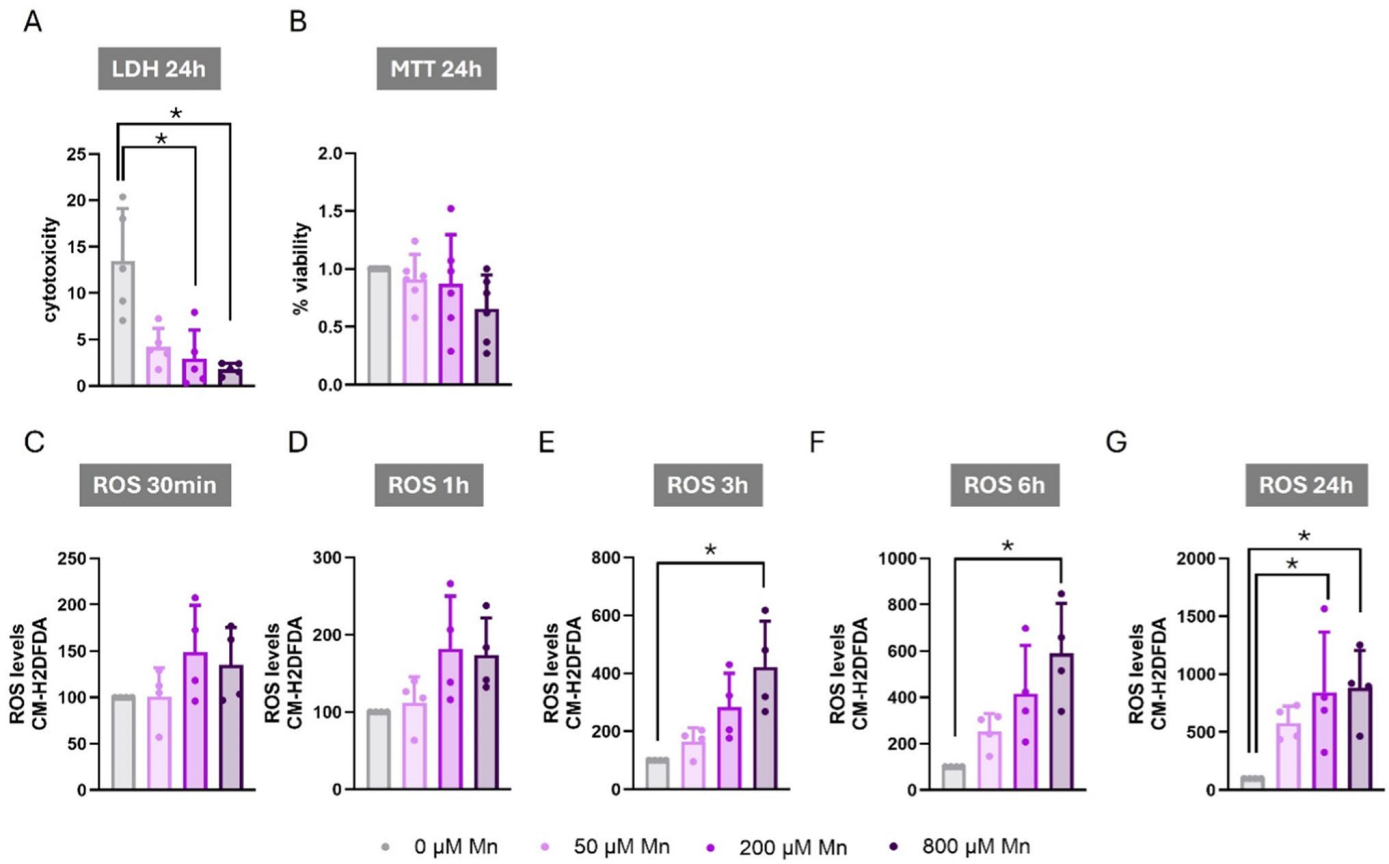
Effects of Manganese Exposure on Cytotoxicity, Cell Viability, and ROS Production in C8-D1A Astrocytic Cells. **A** LDH release after 24 h of exposure to manganese (Mn) at 0, 50, 200, and 800 µM. **B** MTT reduction after 24 h of exposure to Mn at 0, 50, 200, and 800 µM. **C-G** ROS production after exposure to Mn at 0, 50, 200, and 800 µM for (**C**) 30 min, (**D**) 1 h, (**E**) 3 h, (**F**) 6 h, and (**G**) 24 h. Data are presented as mean ± SD. Statistical significance was determined using one-way ANOVA followed by Bonferroni’s post-hoc analysis, or with the Kruskal–Wallis test followed by Dunn’s post hoc test, adjusted by Bonferroni correction when normality was not achieved. *P* < 0.05 was considered statistically significant. * denotes significant difference

To investigate the impact of Mn in astrocytes, we first measured the Mn concentration–response associated with cytotoxicity. C8-D1A astrocytic cells were exposed to 50, 200, or 800 µM Mn for 24 h, and cytotoxicity was assessed using LDH assay. These concentrations are expected to capture the subtoxic to toxicity range observed in mammalian brains [57]. After 24 h, Mn exposure did not result in cell lethality. Rather, Mn-treated cells showed a significant decrease in LDH release (H_(3)_ = 11.503, *p* = 0.009) relative to vehicle (Fig. 1A).

To corroborate these findings, cell viability was evaluated with the MTT Assay. After 24 h of exposure to various Mn concentrations, up to 800 µM, no lethal effects nor increase in survival effects were observed, though the data trended towards a decreased number of viable cells (Fig. 1B).

Despite the absence of cytotoxicity, Mn exposure elicited a significant concentration-dependent increase in ROS production at 3 h (H_(3)_ = 11.194, *p* = 0.011), 6 h (F_(3,12)_ = 7.490, *p* = 0.004), and 24 h (F_(3,12)_ = 5.249, *p* = 0.015) (Fig. 1C, D, E, F, and G).

These results suggested that exposure to Mn increased oxidative stress in C8-D1A astrocytic cells over 24 h, in the absence of cell mortality changes. Based on these findings, we used 200 µM Mn in subsequent co-exposure experiments with Fe.

### Iron Exposure Induced Oxidative Stress in C8-D1A Astrocytic Cells Without Impacting Cell Viability

**Fig. 2:**
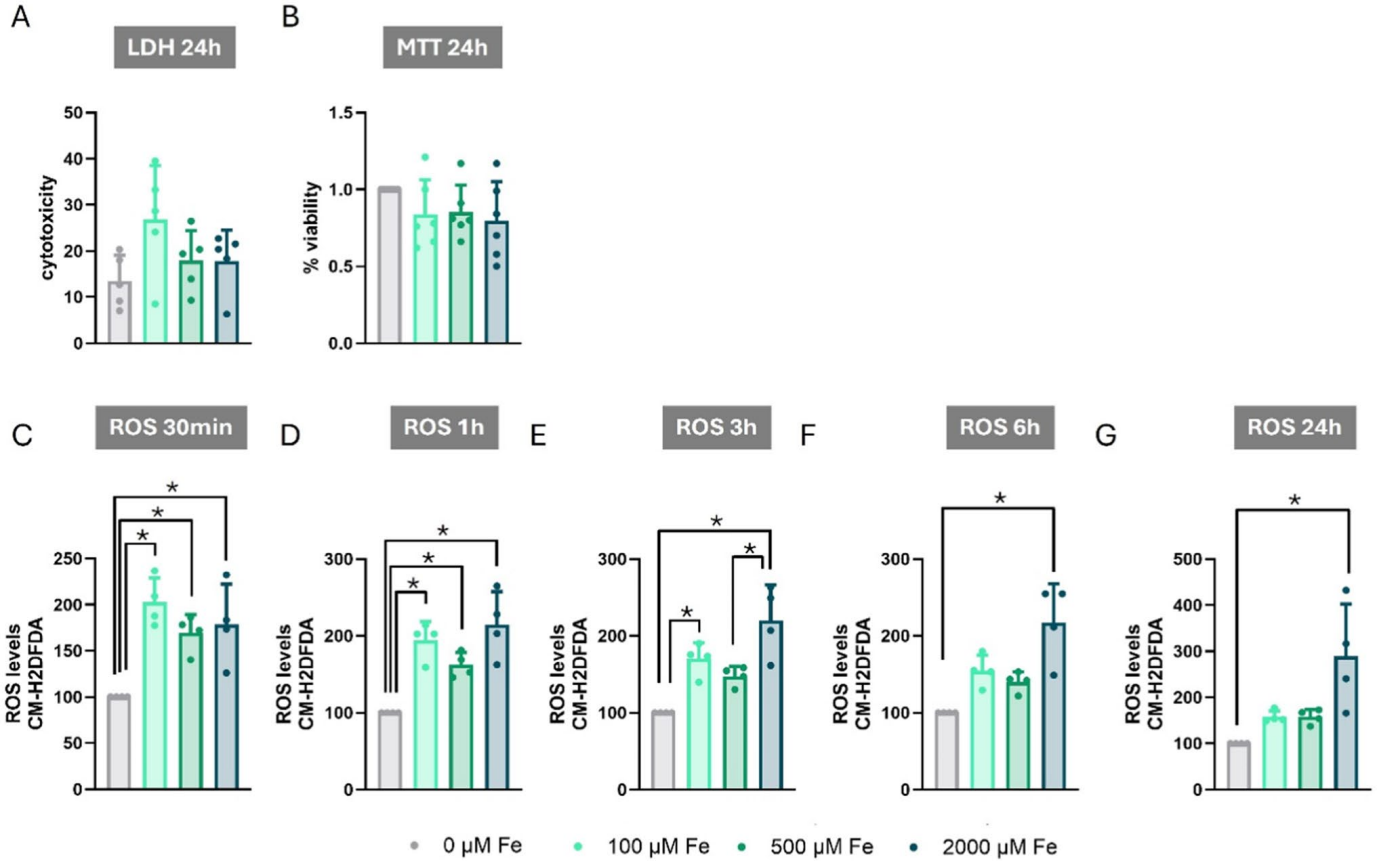
Effects of Iron Exposure on Cytotoxicity, Cell Viability, and ROS Production in C8-D1A Astrocytic Cells. **A** LDH release after 24 h of exposure to iron (Fe) at 0, 100, 500, and 2000 µM. **B** MTT reduction after 24 h of exposure to Fe at 0, 100, 500, and 2000 µM. **C-G** ROS production after exposure to Fe at 0, 100, 500, and 2000 µM for (**C**) 30 min, (**D**) 1 h, (**E**) 3 h, (**F**) 6 h, and (**G**) 24 h. Data are presented as mean ± SD. Statistical significance was determined using one-way ANOVA followed by Bonferroni’s post-hoc analysis, or with the Kruskal–Wallis test followed by Dunn’s post hoc test, adjusted by Bonferroni correction when normality was not achieved. *P* < 0.05 was considered statistically significant. * denotes significant difference

Next, we investigated the effects of Fe exposure on C8-D1A cells by assessing cell viability and oxidative stress. Cell viability was evaluated using LDH and MTT assays after 24 h of exposure to 100, 500, or 2000 µM Fe. No significant changes in cell viability were observed at any of the tested Fe concentrations, as determined by both the LDH and MTT assays (Fig. 2A and B).

Despite the lack of significant cytotoxicity, Fe exposure significantly increased ROS production. This induction was evident as early as 30 min of Fe exposure (F_(3,12)_ = 10.405, *p* = 0.001) (Fig. 2C), and remained detectable at longer exposure times: 1 h F_(3,12)_ = 14.845, *p* < 0.001), 3 h (F_(3,12)_ = 14.602, *p* < 0.001), 6 h (H_(3)_ = 11.821, *p* = 0.008), and 24 h (H_(3)_ = 11.485, *p* = 0.009)) (Fig. 2D, E, F, and G).

Similar to the Mn exposure experiments described previously, Fe exposure induced oxidative stress in C8-D1A cells without altering cell survival at the equivalent tested exposure concentrations.

### Combined Manganese and Iron Exposure Does Not Affect Cell Viability in C8-D1A Astrocytic Cells

**Fig. 3:**
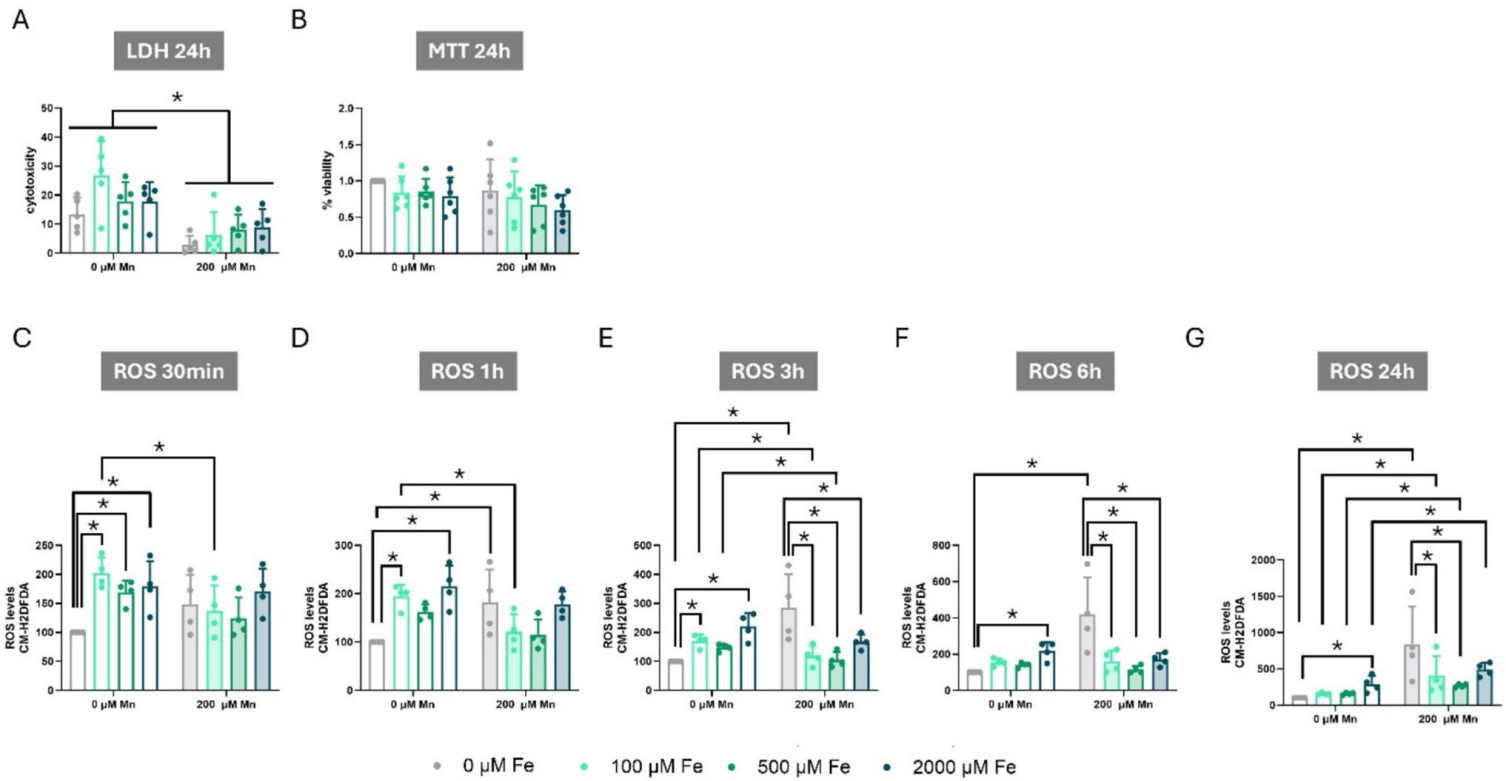
Effects of Combined Manganese and Iron Exposure on Cytotoxicity, Cell Viability, and ROS Production in C8-D1A Astrocytic Cells. **A** LDH release after 24 h of exposure to 200 µM Mn, Fe at 0, 100, 500, and 2000 µM, or a combination of both metals. **B** MTT reduction after 24 h of exposure to 200 µM Mn, Fe at 0, 100, 500, and 2000 µM, or a combination of both metals. **C-G** ROS production after exposure to 200 µM Mn, Fe at 0, 100, 500, and 2000 µM, or a combination of both metals for (**C**) 30 min, (**D**) 1 h, (**E**) 3 h, (**F**) 6 h, and (**G**) 24 h. Data are presented as mean ± SD. Statistical significance was determined using two-way ANOVA followed by Bonferroni’s post-hoc analysis. When normality was not reached, a logarithmic or square root transformation was applied to a better approximate a normal distribution before performing the two-way ANOVA. *P* < 0.05 was considered statistically significant. * denotes significant difference

To investigate the effects of combined Mn and Fe exposure on cell viability, we exposed C8-D1A cells to 100, 500, or 2000 µM Fe alone or in combination with 200 µM Mn. After 24 h, no significant cytotoxicity was observed with Fe exposure, whereas Mn exposure significantly decreased LDH release in all the Fe exposed groups similar to what we observed with Mn alone (F_Mn(1,32)_ = 32.905, *p* < 0.001) (Figs. 1A and 3A). However, and consistent with Mn or Fe alone, this effect was not observed when cell viability was assessed using MTT assay (Figs. 1B, 2B, and 3B). Overall, these results suggest that combined Mn-Fe exposure did not significantly impact cell survival in C8-D1A cells at the concentrations tested.

### Combined Manganese-Iron Exposure Attenuates Oxidative Stress Compared to Manganese or Iron Alone in C8-D1A Astrocytic Cells

Next, we investigated the impact of combined Mn and Fe exposure on oxidative stress by measuring ROS production with CM-H2DCFDA, a whole cell oxidative stress indicator. A significant interaction between Mn and Fe was observed at 30 min (F_Int(3,24)_ = 4.125, *p* = 0.017) (Fig. 3C). While exposure to 200 µM Mn alone did not significantly elevate ROS levels compared to non-exposed cells, Fe exposure increased ROS production at all doses tested (Bonferroni’s post hoc *p* = 0.002 for 100 µM Fe, *p* = 0.044 for 500 µM Fe, and *p* = 0.022 for 2000 µM Fe). Interestingly, when Mn and Fe were co-exposed, Mn exposure only attenuated the Fe-induced ROS production at the lower Fe concentration (100 µM) (Bonferroni’s post hoc *p* = 0.017).

Similarly, a significant interaction was observed at 1 h (F_Int(3,24)_ = 7.295, *p* = 0.001) (Fig. 3D), where 200 µM Mn exposure significantly elevated ROS production compared to control conditions (Bonferroni’s post hoc *p* = 0.004). A similar effect was observed with Fe exposure alone (Bonferroni’s post hoc *p* = 0.007 for 100 µM and *p* < 0.001 for 2000 µM). Interestingly, Mn exposure attenuated the Fe-induced increase in ROS levels at the 100 µM Fe concentration (Bonferroni’s post hoc *p* = 0.008).

At 3 h, a significant interaction between Mn and Fe exposure on ROS production was observed (F_Int(3,24)_ = 16.780, *p* < 0.001) (Fig. 3E). Exposure to 200 µM Mn alone (Bonferroni’s post hoc, *p* < 0.001), and 100 µM Fe (Bonferroni’s post hoc, *p* = 0.021) and 2000 µM Fe (Bonferroni’s post hoc *p* < 0.001) significantly elevated ROS levels compared to non-exposed cells when used alone. Interestingly, combined exposure to Mn and Fe, at all Fe concentrations tested, resulted in a significant attenuation of ROS production compared to 200 µM Mn exposure alone, suggesting a potential ameliorative effect of combined exposure on ROS production.

A significant interaction between Mn and Fe was also observed at 6 h (F_Int(3,24)_ = 15.970, *p* < 0.001) and at 24 h (F_Int(3,24)_ = 7. 474, *p* = 0.001) (Fig. 3F and G). At both time points, exposure to Mn alone significantly elevated ROS production compared to control cells (Bonferroni’s post hoc *p* < 0.001 and *p* < 0.001, respectively). Fe exposure alone at the highest dose tested increased ROS production at 6 and 24 h (Bonferroni’s post hoc *p* = 0.003, in both cases). Notably, co-exposure with Mn and Fe attenuated ROS production compared to cells exposed to Mn alone. However, at 24-h time point, this attenuating effect was not observed with the highest Fe concentration (2000 µM), while significant attenuation of ROS production was seen with the lower Fe concentrations (100 µM (Bonferroni’s post hoc *p* = 0.040) and 500 µM (Bonferroni’s post hoc *p* = 0.003)). Interestingly, at 24 h. Mn exacerbated ROS production induced by 100 µM Fe (Bonferroni’s post hoc *p* = 0.005), 500 µM Fe (Bonferroni’s post hoc *p* = 0.043), and 2000 µM Fe (Bonferroni’s post hoc *p* = 0.032) (Fig. 3G).

Collectively, these data demonstrate that both Mn and Fe alone elevated ROS production. Importantly, co-exposure not only failed to exacerbate oxidative stress, but actually attenuated the increased ROS production. These findings suggest an antagonistic mechanistic interaction between Mn and Fe, as combined exposure resulted in significantly lower ROS levels compared to exposure alone.

Given that both 500 µM Fe and 200 µM Mn concentrations alone induced ROS production, and their antagonistic interaction, evident by the attenuation of oxidative stress, persisted at 24 h, these concentrations were selected for the subsequent experiments.

### Combined Exposure of Manganese and Iron Did Not Affect Glutathione (GSH) Levels in C8-D1A Astrocytic Cells

**Fig. 4:**
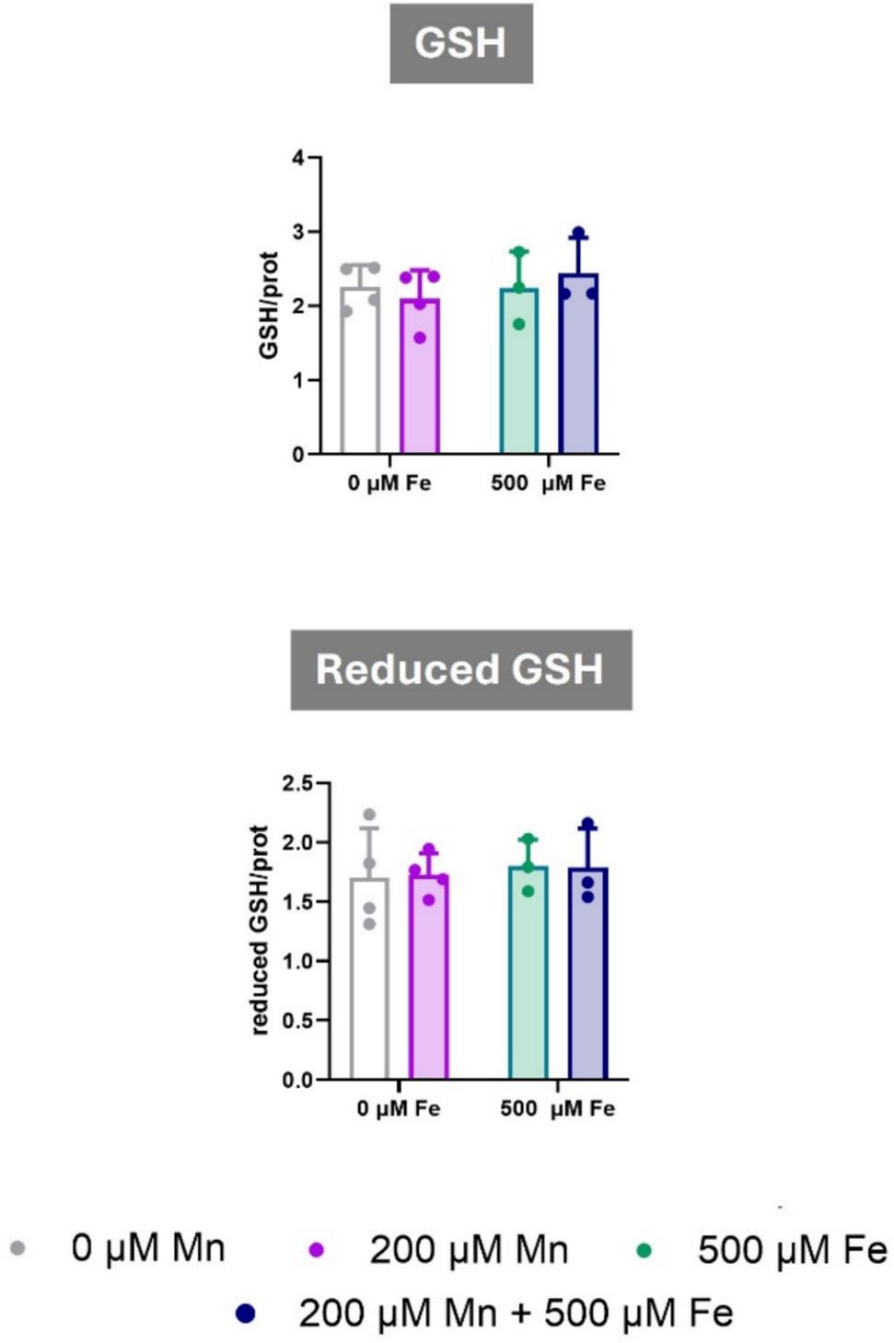
Effects of Combined Manganese and Iron Exposure on glutathione levels in C8-D1A Astrocytic Cells. **A** Total glutathione (GSH) levels after 24 h of exposure to 200 µM Mn, 500 µM Fe, or a combination of both metals. **B** Reduced GSH levels after 24 h of exposure to 200 µM Mn, 500 µM Fe, or a combination of both metals. Data are presented as mean ± SD. Statistical significance was determined using two-way ANOVA followed by Bonferroni’s post-hoc analysis. *P* < 0.05 was considered statistically significant. * denotes significant difference

To elucidate the mechanisms underlying the observed antioxidant antagonism between Mn and Fe, we assessed intracellular GSH levels. GSH is a critical component of the antioxidant defense system regulating redox homeostasis [58]. C8-D1A astrocytic cells were exposed to 200 µM Mn and 500 µM Fe, either alone or in combination. After 24 h of exposure, no significant changes in total or reduced GSH were detected (Fig. 4A and B). These findings suggest that combined Mn-Fe exposure does not significantly alter GSH levels and may reduce ROS through a mechanism independent of GSH.

### Manganese and Iron Synergistically Upregulate Nqo1 Gene Expression, Modulating Antioxidant Pathways in C8-D1A Astrocytic Cells

**Fig. 5:**
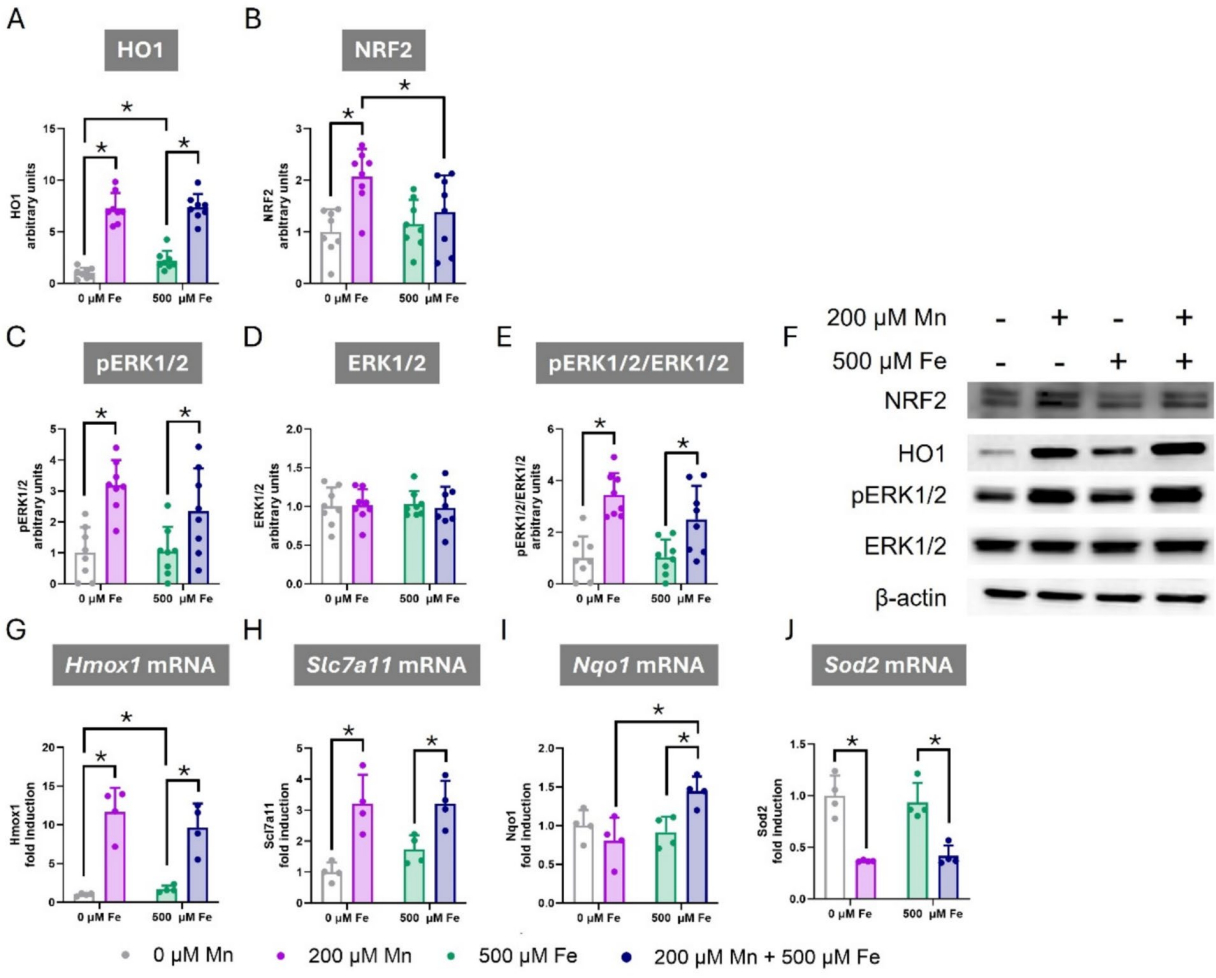
Effects of Combined Manganese and Iron Exposure on Cellular Signaling and Antioxidant Gene Expression in C8-D1A Astrocytic Cells. C8-D1A astrocytic cells were exposed to 200 µM Mn, 500 µM Fe, or a combination of both metals for 24 h. **A-E** Western blot analysis of (**A**) HO1, (B) NRF2, (**C**) phosphorylated ERK1/2 (pERK1/2), (**D**) total ERK1/2, and (**E**) the ratio of pERK1/2 to total ERK1/2 protein (**F**) Representative Western blot images for NRF2, HO-1, pERK1/2, ERK1/2, and β-actin. **G-J** Quantitative PCR analysis of mRNA expression levels for (**G**) *Hmox1*, (**H**) *Scl7a11*, (**I**) *Nqo1*, and (**J**) *Sod2*. Data are presented as mean ± SD. Statistical significance was determined using two-way ANOVA followed by Bonferroni’s post-hoc analysis. When normality was not reached, a logarithmic or square root transformation was applied to a better approximate a normal distribution before performing the two-way ANOVA. *P* < 0.05 was considered statistically significant. * denotes significant difference

Heme oxygenase 1 (HO1) is a crucial enzyme in cellular antioxidant defense system and is induced by Mn exposure as a cytoprotective mechanism [59]. Following 24 h of exposure to 200 µM Mn or 500 µM Fe, either alone or in combination, HO1 protein levels were assessed by western blot. A significant interaction (F_Int(1,28)_ = 6.419, *p* = 0.017) showed that Mn (Bonferroni’s post hoc *p* < 0.001) and Fe (Bonferroni’s post hoc *p* < 0.001) increased HO1 expression when exposed alone. Interestingly, Mn and Fe co-exposure exacerbated HO1 protein levels compared to Fe exposed cells (Bonferroni’s post hoc *p* < 0.001) but not compared to Mn exposed cells (Fig. 5A).

Nuclear factor erythroid 2-related factor 2 (NRF2) is a pivotal transcription factor that orchestrates cellular defense mechanisms by promoting the expression of various antioxidant enzymes, including HO1 [60–64]. A significant interaction between Mn and Fe exposure was observed on NRF2 protein levels (F_Int(1,28)_ = 4.611, *p* = 0.041) (Fig. 5B). Specifically, Mn exposure significantly increased NRF2 protein levels (Bonferroni’s post hoc *p* < 0.001). Interestingly, this effect was attenuated upon co-exposure with Fe (Bonferroni’s post hoc *p* = 0.019), suggesting that the decrease in NRF2 protein levels in Mn and Fe co-exposed cells may reflect a reduction in oxidative stress. This attenuation may reflect a shift towards the restoration of cellular homeostasis, potentially due to a decrease in oxidative stress levels in the presence of both metals, signaling a return to baseline cellular conditions.

Extracellular signal-regulated kinase 1 and 2 (ERK1/2) by regulating NRF2 activity also plays a key role in cellular antioxidant defense [65–67]. In this study, we observed a significant increase in ERK1/2 phosphorylation in response to Mn (F_Mn(1,28)_ = 24.900, *p* < 0.001). In contrast, Fe exposure did not alter ERK 1/2 phosphorylation levels (Fig. 5C). No significant changes in total ERK 1/2 protein expression were detected upon exposure to either Mn or Fe (Fig. 5D). Consistent with the observed changes in ERK1/2 phosphorylation, the ratio phosphorylated ERK 1/2 to total ERK 1/2 was significantly increased only in Mn-treated C8-D1A cells (F_Mn(1,28)_ = 33.561, *p* < 0.001) (Fig. 5E), suggesting that Mn activated ERK 1/2 signaling pathway. The lack of an effect of Fe exposure on the ratio phosphorylated ERK 1/2 to total ERK 1/2 suggests that the antagonistic effects of Mn and Fe on NRF2 protein levels involves distinct signaling mechanisms independent of the ERK 1/2 pathway.

Given NRF2’s role as a transcription factor, we measured the expression of known antioxidant NRF2 target genes. The *Hmox1* gene encodes HO1 protein, an important antioxidant enzyme. Following 24-h exposure to 200 µM Mn or 500 µM Fe, either alone or in combination, a significant interaction in *Hmox1* mRNA expression was observed (F_Int(1,12)_ = 6.821, *p* = 0.028). *Hmox1* gene expression increased in Mn (Bonferroni’s post hoc *p* < 0.001) and in Fe-exposed cells alone (Bonferroni’s post hoc *p* = 0.026) compared with non-treated cells. Notably, Mn and Fe co-exposed cells exacerbated Fe-induced ROS production (Bonferroni’s post hoc *p* < 0.001), while they did not differ from Mn-exposed cells (Fig. 5G), paralleling the changes observed in HO1 protein levels (Fig. 5A).

To further elucidate the mechanisms underlying the observed antagonistic effect of Mn and Fe on ROS production, we measured the expression of additional antioxidant genes using qPCR.

Firstly, we examined Solute carrier family 7 member 11 (*Slc7a11*) gene expression, which encodes a crucial transporter protein for maintaining cellular glutathione levels and managing oxidative stress [68]. In this case, Mn exposure significantly increased *Slc7a11* mRNA expression (F_Mn(1,12)_ = 31.430, *p* < 0.001), whereas Fe exposure had no significant effect (Fig. 5H).

Secondly, we assessed the expression of NAD(P)H Quinone Dehydrogenase 1 (*Nqo1*), an enzyme involved in detoxifying reactive quinones and maintaining redox balance [69]. Neither 200 µM Mn nor 500 µM Fe exposure alone significantly affected the *Nqo1* gene expression compared to control conditions. However, co-exposure to both Mn and Fe resulted in a significant increase in gene expression (F_Int(1,12)_ = 10.151, *p* = 0.008, Bonferroni’s post hoc *p* = 0.002 compared to Mn exposure and *p* = 0.006 compared to Fe exposure) (Fig. 5I) This synergetic interaction indicated that the combination of Mn and Fe leads to an upregulation of *Nqo1* gene expression, despite neither metal having a significant effect individually. This finding support the hypothesis that NQO1 may contribute to the antioxidant effects observed in cells co-exposed to Mn and Fe.

Finally, we investigated Superoxide dismutase 2 (*Sod2*) gene expression, a mitochondrial antioxidant enzyme responsible for converting superoxide radicals into less harmful molecules [70, 71]. Interestingly, 200 µM Mn significantly decreased *Sod2* gene expression (F_Mn(1,12)_ = 85.116, *p* < 0.001), while 500 µM Fe had no significant effect (Fig. 5J). These results support the hypothesis that the Mn-induced increase in ROS production may, at least in part, be mediated by a downregulation of *Sod2* gene expression.

### Manganese Exposure Impairs STAT3 Signaling, with no Additional Effects from Co-Exposure to Iron in C8-D1A Astrocytic Cells

**Fig. 6:**
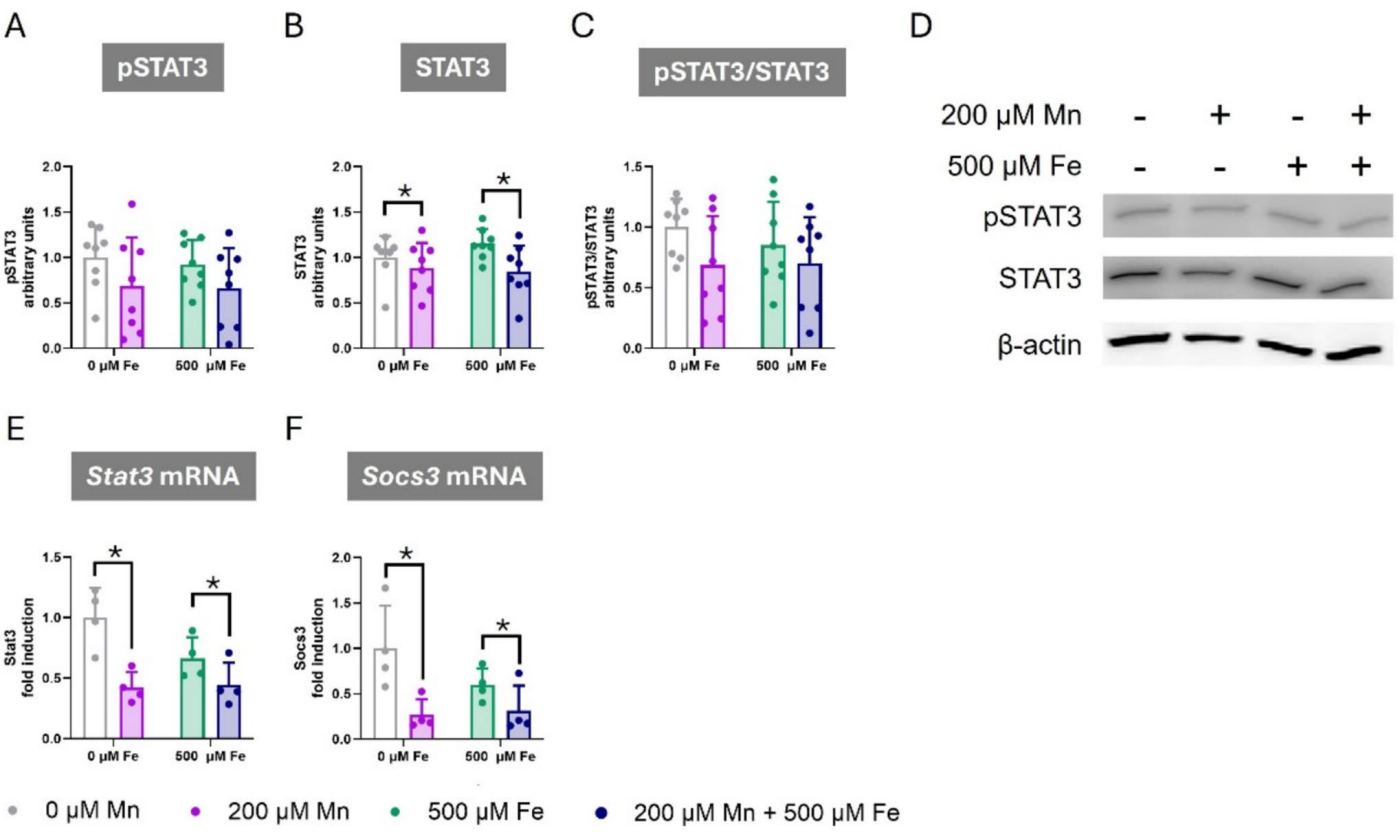
Effects of Combined Manganese and Iron Exposure on STAT3 Cellular Signaling and Gene Expression in C8-D1A Astrocytic Cells. C8-D1A astrocytic cells were exposed to 200 µM Mn, 500 µM Fe, or a combination of both metals for 24 h. **A-C** Western blot analysis of (**A**) phosphorylated STAT3 (pSTAT3), **B** total STAT3, and (**C**) the ratio of pSTAT3 to total STAT3 protein (**D**) Representative Western blot images for pSTAT3, STAT3, and β-actin. **E–F** Quantitative PCR analysis of mRNA expression levels for (**E**) *Stat3*, and (**F**) *Socs3*. Data are presented as mean ± SD. Statistical significance was determined using two-way ANOVA followed by Bonferroni’s post-hoc analysis. When normality was not reached, a logarithmic transformation was applied to a better approximate a normal distribution before performing the two-way ANOVA. *P* < 0.05 was considered statistically significant. * denotes significant difference

Given the observed decrease in *Sod2* gene expression following Mn exposure, we investigated whether the observed changes on cellular redox balance might involve the modulation of Signal Transducer and Activator of Transcription 3 (STAT3) signaling pathway. STAT3 is a transcription factor known to play a critical role in the cellular response to oxidative stress, and it has been demonstrated to regulate *Sod2* gene expression [72]. Following 24-h exposure to 200 µM Mn or 500 µM Fe, either alone or in combination, no effect on STAT3 phosphorylation was observed (Fig. 6A). While Mn exposure reduced total STAT3 protein levels (F_Mn(1,28)_ = 5.728, *p* = 0.024), Fe exposure had no effect (Fig. 6B). Regarding the ratio of phosphorylated STAT3 to total STAT3, no significant effects were detected in C8-D1A cells exposed to Mn, Fe, or a combination of both (Fig. 6C), suggesting that Mn-exposure significantly impaired STAT3 protein levels without affecting phosphorylation levels. To further understand the effects of these metals in STAT3, we assessed the gene expression of *Stat3* and Suppressor of Cytokine Signaling 3 (*Socs3)* using qPCR. After 24-h exposure to 200 µM Mn or 500 µM Fe, either alone or in combination, Mn exposure significantly decreased both *Stat3* (F_Mn(1,12)_ = 18.031, *p* < 0.001) (Fig. 6E) and *Socs3* (F_Mn(1,12)_ = 17.547, *p* = 0.001) (Fig. 6F), while Fe exposure had no significant effect. These findings support the hypothesis that STAT3 signaling pathway is not involved in the antagonistic effects of Mn and Fe. Moreover, they indicate that Mn impairment of STAT3 signaling, is likely mediated by a reduction in total STAT3 protein levels rather than changes in phosphorylation.

## Discussion

In this novel study, we demonstrated that combined exposure to Mn and Fe resulted in a reduction of ROS production compared to exposure to Mn or Fe alone in C8-D1A astrocytic cell line. This reduction in ROS production appears to be mediated through the activation of the NRF2/NQO1 signaling pathway.

Mn and Fe individually have both been independently shown to contribute to the degeneration of dopaminergic neurons within the substantia nigra, which can lead to neurodegenerative diseases such as PD, and to a lesser extent, AD [3, 73–76]. Human exposure to Mn and Fe occurs by various routes, including dietary intake and occupational exposure, highlighting the potential for individuals to be exposed to either Mn, Fe, or both metals simultaneously [77, 78]. Consequently, it is crucial to evaluate the effects of Mn and Fe in both isolated and combined exposures to better understand their toxicological impact and associated health risks.

Astrocytes are crucial for the maintenance of normal CNS function [79], providing essential neuroprotection against various insults, including those arising from disease or environmental exposures, such as Mn and Fe [29]. While astrocytes are critical for CNS homeostasis, they are also susceptible to the neurotoxic effects of Mn and Fe, with both metals disrupting critical astrocytic functions. Furthermore, astrocytes can accumulate both Mn and Fe within the brain, contributing to neurodegenerative processes through the induction of oxidative stress and neuroinflammatory processes [9, 80–84]. Notably, there is a significant gap in our understanding of combined effects of Mn and Fe exposure on the CNS, particularly regarding the role of astrocytes, due to the limited number of studies directly investigating astrocytic responses to combined Mn and Fe exposure.

Mn exposure is known to disrupt cellular redox homeostasis, leading to increased production of ROS and oxidative stress. This has been also observed in astrocytes, where studies show that Mn exposure increased ROS production in rat cortical astrocytic primary cultures [80, 82] and in various astrocytic cell lines [85, 86]. Consistent with these findings, this study also detected an increase in ROS production in C8-D1A cells exposed to Mn. Similarly, Fe exposure induced an increase in ROS levels in our study. Prior research has also demonstrated Fe-induced oxidative stress in rat cortical astrocytic primary cultures [87]. This is consistent with the well-known role of Fe, particularly in its free form, as a catalyst for the Fenton reaction, which generates ROS. However, an unexpected finding emerged when we examined the effects of combined Mn and Fe exposure. In contrast to the individual metal exposures, combined exposure resulted in a significant decrease in ROS levels compared to exposure alone in C8-D1A astrocytic cells. These data suggest an antagonistic interaction between Mn and Fe in terms of their effects on ROS production. Interestingly this antagonistic effect has been previously observed in other cell types, such as microglial BV-2 cells [88] and human neuroblastoma SH-SY5Y cells [89], suggesting that the observed attenuation of oxidative stress due to combined Mn and Fe exposure is not specific to astrocytes but rather represents a general mechanism across different cell types.

Cells possess intricate antioxidant defense systems to counteract the harmful effects of ROS. The NRF2/KEAP1 pathway constitutes a pivotal component of these defense mechanisms. NRF2 is a transcription factor that regulates the expression of numerous antioxidant genes. Under normal conditions, NRF2 remains inactive in the cytoplasm due to its association with KEAP1. However, when oxidative stress occurs, KEAP1 undergoes conformational changes, facilitating NRF2 to translocate to the nucleus. Once in the nucleus, NRF2 binds to the Antioxidant Response Element (ARE) sequence in the promotor regions of target genes, thereby activating the transcription of antioxidant enzymes, including HO1, SLC7A11, NQO1, and SOD [61, 63, 66, 90, 91]. In C8-D1A astrocytic cells, exposure to Mn increased NRF2 protein levels, indicating activation of NRF2-mediated antioxidant response. Notably, this effect was attenuated upon co-exposure with Fe, mirroring the observed decrease in ROS production in cells co-exposed to Mn and Fe, suggesting a reduction in oxidative stress. This attenuation may reflect a shift towards the restoration of cellular homeostasis, potentially due to a decrease in oxidative stress levels in the presence of both metals, signaling a return to baseline cellular conditions.

Furthermore, when we assessed downstream NRF2-regulated antioxidant enzymes, our findings did not show any effect of the combined Mn and Fe exposure on *Slc7a11* and *Sod2* gene expression. However, a synergetic effect was observed in *Nqo1* genes expression. This finding supports a hypothesis where the antagonistic antioxidant effects of Mn and Fe co-exposure may be mediated, at least in part, through the NRF2/NQO1 signaling pathway. Although NRF2/NQO1 pathway is a plausible mechanism, studies assessing NRF2 nuclear localization, and the use of NQO1 pharmacological inhibition or genetic ablation will help to further characterize this mechanism. Interestingly, in human neuroblastoma SH-SY5Y cells a synergetic effect on nuclear NRF2 protein levels was reported when cells were co-exposed to Mn and Fe, which was accompanied by with a synergetic effect on HO1 and NQO1 [89]. This suggests that the mechanism mediating the antagonistic antioxidant effects may vary between different cell types.

Previous studies have demonstrated that Mn exposure activates ERK1/2 signaling pathway in primary astrocytes [92, 93] and it has been suggested that Fe overload may mediate similar effects [94]. Importantly, the ERK1/2 pathway plays a crucial role in regulating NRF2, as ERK1/2 signaling has been shown to promote NRF2 nuclear translocation, facilitating the expression of antioxidant genes [95–97]. In C8-D1A astrocytic cells, Fe exposure did not significantly alter ERK1/2 activation, whereas Mn induced a robust phosphorylation of ERK1/2, suggesting that the synergetic effects of combined Mn and Fe exposure may involve mechanisms independent of ERK1/2 activation. It is important to note that NRF2 nuclear translocation can be influenced by other signaling pathways in various cellular contexts, including c-Jun N-terminal Kinase (JNK) [98], p38 Mitogen-Activated Protein Kinase (P38) [99], or Phosphoinositide 3-Kinase (PI3K)/Protein Kinase B (AKT) [100]. Therefore, we cannot rule out the possibility that one or more of these pathways are mediating the observed effects of combined Mn and Fe exposure in C8-D1A. Despite the lack of a Fe-induced effect on ERK1/2 phosphorylation in our model, the robust Mn-induced ERK1/2 activation, coupled with the observed increase in antioxidant gene expression, suggests that ERK1/2 may still contribute, at least in part, to the Mn-induced activation of the NRF2-mediated antioxidant defense.

While Fe exposure alone led to a significant increase in ROS levels at early time points compared to Mn exposure alone, Mn exhibited a more pronounced oxidative stress effect than Fe or combined Mn and Fe exposures. Mn also triggered a more robust activation of the antioxidant defense genes *Hmox1* and *Slc7a11*, which, together with the increase in NRF2 and HO1 protein levels, suggest that Mn exposure activates NRF2-mediated antioxidant responses in C8-D1A astrocytic cells. Similar findings have been reported in neuroglial cells, where Mn exposure induced NRF2 and HO1 [59, 101, 102].

Interestingly, Mn exposure reduced *Sod2* gene expression in C8-D1A astrocytic cells. Literature regarding the impact of Mn on SOD2 is complex. Some studies have reported that Mn increases SOD2 protein levels in primary astrocytes [103, 104], while other have shown that Mn decreases SOD activity in neuroglial cells [89, 92, 103]. Although *Sod2* mRNA expression was decreased in C8-D1A cells, this is not necessarily incompatible with increased protein levels in the cytoplasm. Furthermore, a Mn decrease in SOD activity could occur via direct or indirect mechanisms, Further investigations are needed to clarify this discrepancy.

In C8-D1A astrocytic cells, Mn exposure downregulated *Sod2* gene expression and induced Nrf2 protein expression. While Nrf2 is a key regulator of antioxidant genes, SOD2 expression is also influenced by other pathways, including the STAT3 signaling pathway. STAT3, a transcription factor crucial for diverse cellular functions, including cell survival, cell growth, and inflammation, plays a significant role in maintaining redox homeostasis [105, 106]. It downregulates the expression of certain mitochondrial proteins [107] and upregulates antioxidant gene expression, including SOD2 [72], reducing intracellular ROS levels. Notably, our findings indicate that Mn exposure inhibited STAT3. Given the observed *Sod2*, downregulation and the known role of STAT3 in regulating *Sod2* expression, it is plausible that the Mn-mediated oxidative stress, at least in part, may be attributed to the inhibition of STAT3 and the consequent downregulation of *Sod2*.

The protective effect observed during Mn and Fe co-exposure may also be explained by the Fe’s higher affinity for shared transporter proteins. Mn and Fe share several transporter proteins, such as DMT1 [34], which facilitates their uptake into the cells, and FPN [37], which facilitates their efflux from cells. While they compete for these transporters, Fe has a higher affinity for them. Fe’s affinity for FPN has been reported to be three orders of magnitude greater compared with Mn [108]**.** Consistent with this, Fe supplementation has been shown to decrease Mn accumulation in animals and human studies [109–111]. Furthermore, epidemiological studies showed that individuals with Fe deficiency have higher Mn blood levels [42, 44, 112–114]**.** Conversely, high Mn levels inhibit Fe absorption [115, 116]. Notably, Mn supplementation can increase Fe accumulation in animals fed regular or Fe-deficient diets [117]**,** although this effect appears to be cell type dependent [118]. Taken together, these findings suggest that the Mn and Fe competition for the transporters may confer neuroprotection. In our study, it is plausible that the Fe’s higher affinity for DMT1 and FPN reduced Mn uptake and efflux, thereby decreasing Mn accumulation and protecting the cells. Further investigations into the cellular accumulation of Mn and Fe are needed to clarify this mechanism.

### Limitations

This study utilized C8-D1A astrocytes for experimentation. However, C8-D1A astrocytes are type I clones, meaning they are cultured cell lines rather than primary cells obtained from living organisms. While the use of an immortalized cell line provides a controlled environment for mechanistic studies, it is crucial to acknowledge inherent limitations. In vitro models lack the complexity of the in vivo environment, potentially limiting the direct translation of findings to physiological conditions. Consequently, results derived from C8-D1A cells may not fully replicate the oxidative stress processes that occur in vivo.

In our study, we did not measure the accumulation of Mn and Fe in the cells. This has to be taken into account in the interpretation of the results. Manganese accumulation is neurotoxic, the higher Fe’s affinity for the transporter proteins may reduce Mn uptake protecting cells.

The NRF2/NQO1 pathway offers a plausible mechanism to explain the antagonistic effects of Mn and Fe. However, a more comprehensive understanding would be achieved through studies analyzing NRF2 nuclear localization and using NQO1 pharmacological inhibitors or genetic ablation.

Despite these limitations, this study contributes valuable insights into the interplay of Mn and Fe in inducing oxidative stress in astrocytes, a critical area with limited prior research.

## Conclusions

In conclusion, this novel study investigated the effects of combined Mn and Fe exposure on C8-D1A astrocytic cells. Our results demonstrated a significant reduction in ROS levels in cells exposed to both metals compared to individual metal exposures. The magnitude of oxidative stress directly correlates with the extent of NRF2 activation. Therefore, the antagonistic effect of combining Mn and Fe initially reduces oxidative stress, which is reflected in the NRF2 response. Additionally, we observed a significant upregulation of *Nqo1* gene expression in the combined exposure group. These findings support a hypothesis that the observed reduction in ROS levels may be mediated, at least in part, by the upregulation of NQO1, highlighting the complex interaction between Mn and Fe in modulating oxidative stress response. However, further research is needed to fully elucidate the underlying mechanisms. Moreover, in vivo verification of these results is critical in extending these findings to human conditions. A comprehensive understanding of these mechanisms could provide valuable insights into the role of metal-induced oxidative stress in neurodegenerative diseases such as PD.

## Data Availability

No datasets were generated or analysed during the current study.
